# Prostatic incidental uptake on PET/CT: an updated systematic review and meta-analysis of prevalence and malignancy risk

**DOI:** 10.3389/fonc.2026.1872033

**Published:** 2026-06-22

**Authors:** Chiara Martinello, Cesare Michele Iacovitti, Andreea Marin, Slavko Tasevski, Alessio Rizzo, Marco Cuzzocrea, Gaetano Paone, Domenico Albano, Giorgio Treglia

**Affiliations:** 1 Division of Nuclear Medicine, Imaging Institute of Southern Switzerland, Ente Ospedaliero Cantonale, Bellinzona, Switzerland; 2 Clinic of Nuclear Medicine, Central University Emergency Military Hospital “Dr. Carol Davila”, Bucharest, Romania; 3 University Institute for Positron Emission Tomography, Skopje, North Macedonia; 4 Department of Nuclear Medicine, Candiolo Cancer Institute, FPO-IRCCS, Turin, Italy; 5 Faculty of Biomedical Sciences, Università della Svizzera, Lugano, Italiana, Switzerland; 6 Nuclear Medicine Department, ASST Spedali Civili, University of Brescia, Brescia, Italy; 7 Faculty of Biology and Medicine, University of Lausanne, Lausanne, Switzerland

**Keywords:** [18F]FDG PET/CT, incidental prostatic uptake, malignancy risk, meta-analysis, prostate cancer, prostate incidentaloma, systematic review

## Abstract

**Background:**

Prostatic incidental uptake (PIU) may have clinical relevance, and progress in molecular imaging is likely to increase its detection. This study aimed to provide updated pooled estimates of the prevalence and malignancy risk of PIU identified on [18F]FDG positron emission tomography/computed tomography (PET/CT) and on PET/CT scans using radiotracers other than [18F]FDG.

**Methods:**

A comprehensive search for studies on PIU was performed in two databases, screening all available literature up to 30 October 2025. Only original articles reporting PIU were included. A proportion meta-analysis was conducted on a patient-based analysis using a random-effects model to calculate pooled prevalence and malignancy rates. In addition, several variables were evaluated as potential predictors of malignant PIU.

**Results:**

Twenty-four studies met the inclusion criteria. PET/CT was performed with [18F]FDG in 23 studies and with radiolabeled somatostatin analogues (SSA) in one study. The pooled prevalence of PIU was 1.7% for [18F]FDG PET/CT, whereas the prevalence observed in the single eligible SSA PET/CT study was 4.5%. The pooled malignancy rates among PIU cases that underwent further evaluation or biopsy were 21.3% and 59.7%, respectively; no malignant lesions were reported in the SSA study. Malignant PIUs showed a higher mean age and a higher mean SUVmax compared with benign PIUs. A peripheral location of PIU emerged as a predictor of malignancy.

**Conclusions:**

PIU is detected in approximately 1.7% of [18F]FDG PET/CT scans in men and is associated with a relevant risk of malignancy. Accordingly, PIU should prompt individualized clinical correlation, particularly when uptake is focal, peripherally located, or associated with elevated PSA or other suspicious prostate-directed findings. More well-designed studies are required to better define the clinical impact of PIU and to determine its prevalence and clinical relevance when using PET tracers other than [18F]FDG.

## 1 Introduction

Incidental imaging findings, or incidentalomas, are lesions detected on examinations performed for indications unrelated to the organ in which they are identified. Their frequency has increased with the broader use of high-resolution imaging and routine whole-body PET/CT. In many cases, these findings are benign, but when they are not, their relevance depends on anatomical site, imaging modality, and patient characteristics (1, 2).

In nuclear medicine, PET has become an integral component of oncologic and non-oncologic imaging. When combined with CT, PET/CT offers simultaneous assessment of functional information and anatomical details. [18F]FDG is the most widely used PET tracer because it reflects glucose metabolism, a process upregulated in many tumors as well as in inflammatory and infectious conditions (3, 4). Recent reviews have further highlighted the central role of [18F]FDG PET in cancer diagnosis, staging, treatment monitoring, and the assessment of tumor metabolism, while also underscoring the growing interest in radiomics-based applications (5). This lack of specificity means that focal [18F]FDG uptake outside the primary lesion must be interpreted in the clinical context. In addition, other PET tracers beyond [18F]FDG are increasingly used in oncological imaging. Incidental uptake in various organs has also been reported in these scans (1, 2).

In the pelvic region, incidental focal uptake in the prostate has been observed on [18F]FDG PET/CT, even though the gland is not the primary target of the examination (6). In most cases, these scans are performed for non-prostatic malignancies or other systemic indications, and the prostatic uptake is detected post-hoc by the interpreting physician. Similar patterns of uptake can be seen in benign prostatic hyperplasia, prostatitis, granulomatous inflammation, and treatment-related changes, as well as in prostate cancer, which complicates differential diagnosis (6).

In everyday practice, clear estimates of prevalence and malignancy risk of prostatic incidental uptake at PET/CT are essential not only for nuclear medicine physicians but also for urologists, oncologists, and other clinicians who manage patients with these findings.

In this context, we conducted an updated systematic review and meta-analysis to estimate the prevalence of incidental prostatic uptake on PET/CT and the pooled risk of malignancy associated with this finding adding more recent data compared to a previous meta-analysis (6). We also aimed to summarize the main imaging and clinical features related to malignant vs benign uptake and to provide a practical framework for the interpretation and management of incidental prostatic uptake in routine clinical practice.

## 2 Materials and methods

### 2.1 Review protocol, working group, and review question

This systematic review adhered to a prespecified protocol (7), which is provided in full as Supplementary Material (Protocol S1), and was reported following PRISMA 2020 guidelines (8). PROSPERO registration was not pursued, in line with PRISMA recommendations where registration remains optional (8).

Screening and study selection were conducted independently by three reviewers, with discrepancies resolved through discussion involving senior investigators. The primary review question was formulated in advance: “What is the prevalence and clinical relevance of incidental prostate uptake (PIU) detected on PET/CT across different radiotracers?”.

### 2.2 Search strategy

We performed an extensive literature search across PubMed/MEDLINE and Cochrane Library databases up to 30 October 2025. The search strategy integrated terms related to the target organ, incidental detection, and imaging technique: (A) “prostate” OR “prostatic” AND (B) “incidental*” OR “incidentaloma*” OR “unexpected” OR “unusual” AND (C) “PET” OR “positron”.

No restrictions on language or publication date were imposed. Reference lists from included studies and pertinent reviews were manually reviewed to identify further relevant publications.

### 2.3 Study selection

Predefined inclusion and exclusion criteria were applied. Eligible studies included original research articles documenting the prevalence and/or clinical outcomes of PIU on PET/CT performed for non-prostate primary indications using various radiotracers. Excluded were studies outside the topic scope, those without extractable prevalence or malignancy data, narrative reviews, editorials, letters, comments, and single case reports.

Titles and abstracts from the search were independently evaluated by three reviewers. Potentially eligible full-text articles underwent detailed assessment, with final selections determined by consensus. All eligible studies were included in the systematic review. Studies providing sufficient extractable data for at least one quantitative outcome were included in the corresponding outcome-specific meta-analyses.

### 2.4 Data extraction and quality assessment

Data extraction was carried out independently by three reviewers using structured templates capturing: study demographics; radiotracer employed; patient cohort details; total PET/CT scans; overall PIU count; histologically evaluated PIUs (PIU-H); malignant PIUs; histopathological classifications (malignant/benign); and key clinical parameters (SUVmax, PSA levels, age, uptake location, calcifications, prostate volume). Consensus resolved any inconsistencies. Study quality and risk of bias were appraised using the NIH Quality Assessment Tools tailored to study design (9).

### 2.5 Statistical analysis

Outcome-specific random-effects meta-analyses were performed to account for inter-study heterogeneity. Pooled proportions with 95% confidence intervals were calculated for prevalence and malignancy outcomes, standardized mean differences (SMDs) for continuous variables (e.g., SUVmax, PSA, and age), and risk ratios (RRs) for dichotomous variables (e.g., uptake location and calcification pattern). Heterogeneity was assessed using the I² statistic and visual inspection of forest plots. Sensitivity analyses omitted studies showing clear selection bias from quantitative synthesis while retaining them qualitatively. Studies were excluded from outcome-specific meta-analyses when the denominator was not extractable, when only selected or enriched lesions were reported, or when the cohort was clearly not representative of an unselected PET/CT population; these studies were retained in the qualitative synthesis when clinically informative. Statistical computations were performed using MetaAnalysisOnline web platform (https://metaanalysisonline.com/, accessed 30 October 2025).

## 3 Results

### 3.1 Literature search

**Figure 1:**
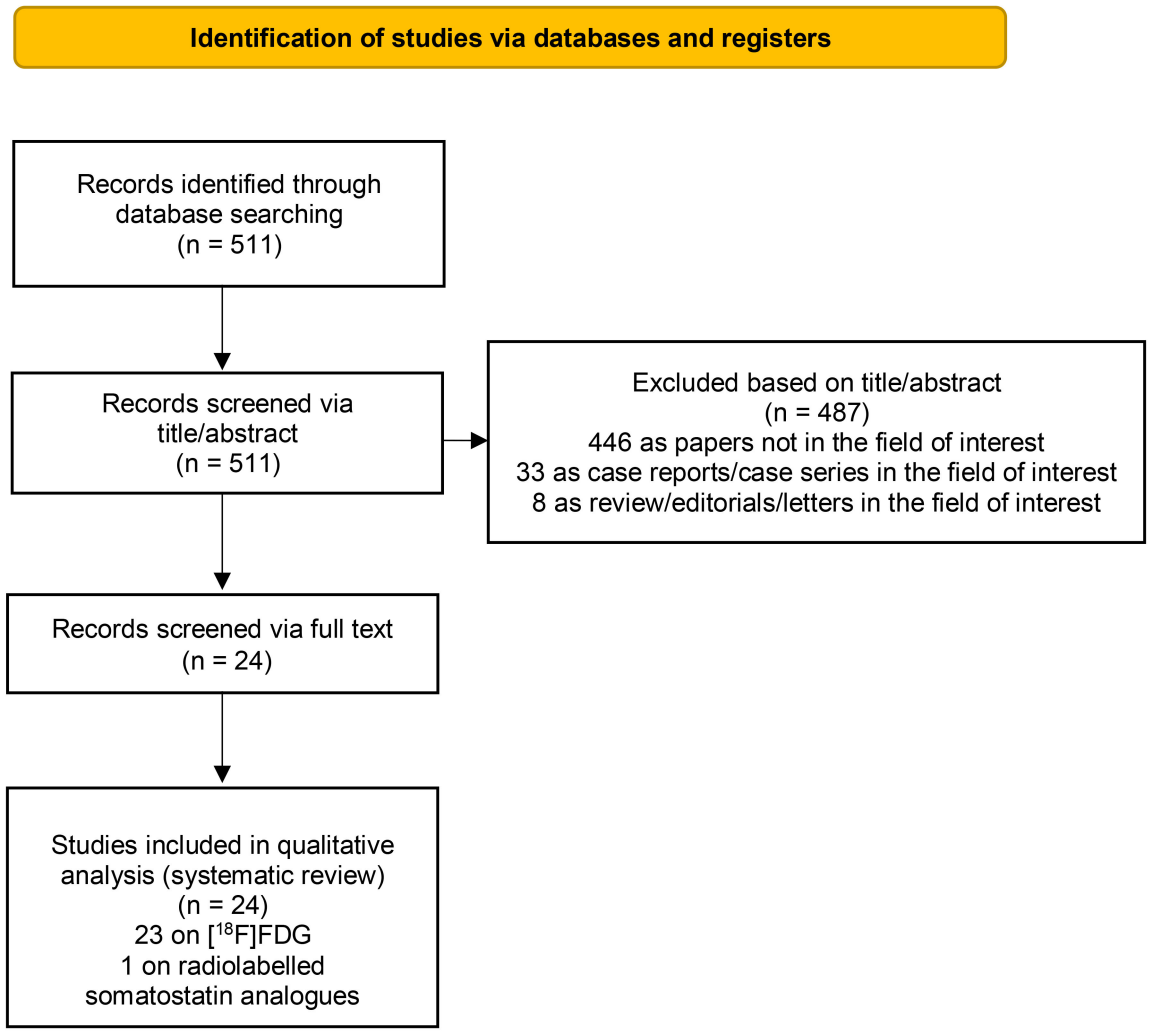
PRISMA 2020 flow diagram for new systematic reviews which included searches of databases and registers only.

The study selection process is outlined in **Figure 1**. The search performed in PubMed/MEDLINE and the Cochrane Library identified the studies potentially relevant to incidental prostatic uptake (PIU) detected on PET/CT. After title/abstract screening, and full-text assessment, 24 articles fulfilled the inclusion criteria for the present systematic review (10–33).

Of these, 23 studies investigated PIU detected on [18F]FDG PET/CT (10–32), whereas one study assessed incidental prostatic uptake on somatostatin receptor PET/CT (33). The quantitative synthesis was mainly based on the [18F]FDG PET/CT literature, while studies with selection bias or without an extractable denominator for prevalence calculation were retained only for the narrative synthesis, according to the predefined methodological criteria. Overall, most included studies were retrospective and single-center in design. Studies such as Brown et al. (18) and other cohorts with clearly selected or enriched populations were retained for narrative synthesis but excluded from prevalence pooling because they did not provide a representative denominator for unselected PET/CT examinations.

### 3.2 Qualitative synthesis

The main characteristics of the included studies, together with the principal data on prevalence, further evaluation, histology, and malignancy rate, are summarized in **Table 1**. In the present review, PIU was considered an incidental prostatic tracer uptake detected on PET/CT examinations performed for indications unrelated to primary prostate disease, although the exact operational definition and the downstream diagnostic work-up were not entirely uniform across studies (10–32).

**Table 1:** Prevalence, malignancy rate and results of biopsy of prostate incidental uptake detected by PET/CT with different tracers.

PET tracer	First author [ref.]	Year	Country/ study type	No. of patients	Patients with PIU (%)	PIU-FE	PIU-H	MPIU-H (%)	Prevalence of MPIU among PIU-FE (%)	ADK (%)	OUM	BPH (%)	OBF
**[^18^F]FDG**	Han et al. (10)	2010	Korea/R	5119	63/5119 (1.2)	55	8	3/8 (37.5)	3/55 (5.4)	3/8 (37.5)	–	NR	NR
	Cho et al. (11)	2011	Korea/R	14854	148/14854 (1)	67	14	9/14 (64.3)	9/67 (13.4)	9/14 (64.3)	–	NR	NR
	Hwang et al. (12)	2013	Korea/R	12037	184/12037 (1.5)	120	38	23/38 (60.5)	23/120 (19.2)	23/38 (60.5)	–	15/38 (–)	–
	Bhosale et al. (13)	2013	USA/R	1440	65/1440 (4.5)	53	26	15/26 (57.7)	15/53 (28.3)	15/26 (57.7)	–	7/26 (–)	4
	Seino et al. (14)	2014	Japan/R	3236	53/3236 (1.6)	49	12	8/12 (66.7)	8/49 (16.3)	7/12 (--)	1	NR	NR
	Yang et al. (15)	2014	China/R	11239	198/11239 (1.8)	100	23	18/23 (78.3)	18/100 (18)	18/23 (78.3)	–	2/23 (--)	3
	Kang et al. (16)	2014	S. Korea/R	18393	106/18393 (0.6)	66	15	11/15 (73.3)	11/66 (16.7)	11	–	4	–
	Kwon et al. (17)	2015	S. Korea/R	47109	1335/47109 (2.8)	99	99	47/99 (47.5)	47/99 (47.5)	41	6	NR	NR
	Bertagna et al. (18)	2015	Italy/R	20422	280/20422 (1.4)	63	63	35/63 (55.6)	35/63 (55.6)	35	–	19	9
	Brown et al. (19) *	2015	USA/R	17	17/17 (100)	17	17	14/17 (82.4)	14/17 (82.4)				
	Sahin et al. (20)	2015	Turkey/R	5985	79/5985 (1.3)	63	12	8/12 (66.7)	8/63 (12.7)	8	–	4	–
	Reesink et al. (21)**	2016	Netherlands/R	108	43/108 (39.8)	43	43	13/43 (30.2)	13/43 (30.2)	NR	NR	NR	NR
	Kim et al. (22)**	2016	S. Korea/R	395	55/395 (13.9)	NR	6	3/6 (50)	NC	3	NR	3	NR
	Conrad et al. (23)**	2016	Germany/R	21	2/21 (9.5)	1	1	1/1 (100)	1/1 (100)	1	–	–	–
	Lee et al. (24)**	2017	S. Korea/P	51	31/51 (60.8)	31	7	4/7 (57.1)	4/31 (12.9)	NR	NR	NR	NR
	Makis et al. (25)	2017	Canada/R	3122	65/3122 (2.1)	53	11	4/11 (36.4)	4/53 (7.5)	4	–	2	5
	Chetan et al. (26)	2017	UK/R	2846	46/2846 (1.6)	18	2	0/2 (0)	0/18 (0)	–	–	–	2
	Mannas et al. (27)	2020	Canada/R	31019	309/31019 (1)	197	47	34/47 (72.3)	34/197 (17.3)	34	–	–	–
	Şahin et al. (28)	2021	Turkey/R	4723	107/4723 (2.3)	83	29	15/29 (51.7)	17/83 (20.5)	NR	NR	NR	NR
	Lee et al. (29)	2022	S. Korea/R	8800	69/8800 (0.8)	39	39	22/39 (56.4)	22/39 (56.4)	22	–	NR	NR
	Nieri et al. (30)**	2023	Italy/R	NR	56 (NC)	45	31	18/31 (58.1)	18/45 (40)	18	–	11	2
	Franklin et al. (31)	2023	Australia/R	9122	273/9122 (3)	231	57	51/57 (89.5)	70/231 (30.3)	51	2		
	Şam Özdemir et al. (32) *	2024	Turkey/R	NR	92 (NC)	30	30	17/30 (56.7)	17/30 (56.7)	17	–	NR	NR
Pooled values (95%CI)				**200058**	**1.7%** **(1.2-2.1)**			**59.7%** **(50.8-68.4)**	**21.3%** **(15-28.2)**				
**Radiolabeled somatostatin analogues**	Gossili et al. (33)	2023	Denmark/R	178	8/178 (4.5)	8	3	0/3 (0)	0/8 (0)	–	–	2	1

ADK, adenocarcinoma; BPH, benign prostatic hyperplasia; NR, not reported; NC, not calculable; PIU, prostate incidental PET/CT tracer uptake; PIU-H, number of prostate incidentalomas that underwent histology; PIU-FE, number of prostate incidentalomas further evaluated; MPIU-H, number of malignancies between prostate incidentalomas that underwent histology; OBF, other or unspecified benign finding (e.g. prostatitis); OUM, other or unspecified malignant lesions; P, prospective; R, retrospective, [18]FDG, 18 fluorine fluorodeoxyglucose.

Studies marked with * or ** were retained in the qualitative synthesis but excluded from specific pooled analyses because of preselected/enriched cohorts, absence of an extractable full denominator, or incomplete outcome reporting.

Substantial inter-study variability was observed with regard to referral indications, sample size, selection criteria, proportion of lesions undergoing additional assessment, and criteria used to establish benignity or malignancy. Histological confirmation was available only in a subset of PIUs in most cohorts, whereas other studies relied on PSA, imaging correlation, or clinical follow-up as part of the diagnostic verification pathway (10–32). This variability should be taken into account when interpreting both prevalence estimates and malignancy rates.

#### 3.2.1 [18F]FDG PET/CT

The evidence on PIU was dominated by [18F]FDG PET/CT, which accounted for 23 of the 24 included studies. Reported PIU prevalence varied widely across cohorts, ranging from less than 1% in large unselected retrospective series to markedly higher values in studies enriched by referral pattern, predefined lesion selection, or dedicated secondary assessment. For this reason, not all studies were suitable for every pooled analysis.

In particular, studies based on selected subgroups rather than on the full denominator of PET/CT examinations were not entered into prevalence meta-analysis, in order to avoid distortion of pooled estimates. This applied, for example, to studies focused on previously identified incidental lesions or on patients already referred for further prostate work-up. These studies remained clinically informative and were therefore retained in the qualitative synthesis, especially for lesion characterization and malignancy profiling.

Among the [18F]FDG-based cohorts, the proportion of malignant lesions among verified PIUs remained substantial, although variable across studies. Prostate adenocarcinoma represented the predominant malignant diagnosis, whereas benign prostatic hyperplasia, prostatitis/granulomatous inflammation, and other nonspecific benign findings were the most frequent non-malignant explanations of uptake. Lesion-level variables reported across studies (**Tables 2**, **3**) included SUVmax, PSA, age, uptake topography, calcification pattern, and prostate volume; these were subsequently explored in the quantitative synthesis when sufficient data were available.

**Table 2:** Characteristics of malignant prostatic incidentalomas at PET/CT.

PET tracer	First author [ref.]	No. of PIU classified as malignant	Mean			Site of tracer uptake		Calcifications			Prostate volume (ml)	
			SUVmax	PSA level (ng/ml)	Age (years)	Peripheral (%)	Central (%)	Concordant (%)	Discordant (%)	Absent (%)	<30 (%)	≥30 (%)
[^18^F]FDG	Han et al. (10)	3	3.1 ± 0.7	39.7 ± 52.6	75 ± 2.6	3/3 (100)	0/3 (0)	0/3 (0)	2/3 (66.7)	1/3 (33.3)	2/3 (66.7)	1/3 (33.3)
	Cho et al. (11)	13	5.5 ± 3.1	33.4 ± 37.9	69.5 ± 8.2	11/13 (84.6)	2/13 (15.4)	3/13 (23.1)	10/13 (76.9)	0/13 (0)	NR	NR
	Hwang et al. (12)	23	5.7 ± 5.1	49.7	73	NR	NR	NR	NR	NR	NR	NR
	Bhosale et al. (13)	15	8.0 ± 6.0	10.2 ± 8.7	72.7 ± 10.0	NR	NR	NR	NR	NR	NR	NR
	Seino et al. (14)	8	7.2 ± 2.0	37.9 ± 31.2	69.7 ± 8.3	8/8 (100)	1/8 (12.5)	0/8 (0)	2/8 (25)	6/8 (75)	NR	NR
	Yang et al. (15)	20	5.6 ± 2.5	NR	66.3 ± 11.1	19/20 (95)	1/20 (5)	4/20 (20)	3/20 (15)	13/20 (65)	10/20 (50)	10/20 (50)
	Kang et al. (16)	11	10.1	127.4	71.6	10 (F)	1 (D)	8/11 (72.7)	NR	NR	3/11 (27.3)	8/11 (72.7)
	Kwon et al. (17)	41	6.9 ± 5.2	8.1 ± 4.8	69.6 ± 8.3	38 (F)	3 (D)	NR	NR	NR	NR	NR
	Bertagna et al. (18)	35	6.8 ± 5.2	7.8 ± 8.2	69.8 ± 11	27/35 (77.1)	8/35 (22.9)	9/35 (25.7)	26/35 (74.3)	–	19/35 (54.3)	16/35 (45.7)
	Brown et al. (19)	–	–	–	–	–	–	–	–	–	–	–
	Sahin et al. (20)	8	4.7 ± 2.3	47.6 ± 24.4	62.6 ± 4.1	6/8 (75)	1/8 (12.5)	NR	NR	NR	NR	NR
	Reesink et al. (21)	13	NR	NR	NR	NR	NR	NR	NR	NR	NR	NR
	Kim et al. (22)	3	NR	NR	NR	NR	NR	NR	NR	NR	NR	NR
	Conrad et al. (23)	1	11	4.9	57	1/1 (100)	–	NR	NR	NR	NR	NR
	Lee et al. (24)	NR	NR	NR	NR	NR	NR	NR	NR	NR	NR	NR
	Makis et al. (25)	4	7.2 ± 2.5	9.2 ± 3.1	74.0 ± 6.8	4/4 (100	–	NR	NR	NR	NR	NR
	Chetan et al. (26)	0	–	–	–	–	–	–	–	–	–	–
	Mannas et al. (27)	34	9.83	14.9	71.8	NR	NR	NR	NR	NR	NR	NR
	Şahin et al. (28)	17	10.6 ± 4.4	17.1	70.4 ± 8.2	NR	NR	NR	NR	NR	NR	NR
	Lee et al. (29)	22	NR	NR	74.1 ± 7.7	NR	NR	NR	NR	NR	NR	NR
	Nieri et al. (30)	18	9 ± 10.6	13.1 ± 14.5	71.9 ± 9.8	NR	NR	NR	NR	NR	NR	NR
	Franklin et al. (31)	70	6.1	9.6	75	61/70 (87.1)	NR	NR	NR	NR	NR	NR
	Şam Özdemir et al. (32)	17	5.5	8.4	69	11 (F)	6 (D)	NR	NR	NR	NR	NR
Radiolabeled somatostatin analogues	Gossili et al. (33)	0	–	–	–	–	–	–	–	–	–	–

D, diffuse; F, focal; NR, not reported; NC, not calculable; SUVmax, maximal standardized uptake value.

**Table 3:** Characteristics of benign prostatic incidentalomas at PET/CT.

PET tracer	First author [ref.]	No. PIU classified as benign	Mean			Site of tracer uptake		Calcifications			Prostate volume (ml)	
			SUVmax	PSA level (ng/ml)	Age (years)	Peripheral (%)	Central (%)	Concordant (%)	Discordant (%)	Absent (%)	<30 (%)	≥30 (%)
[^18^F]FDG	Han et al. (10)	52	3.2 ± 1.7	NR	NR	35/52 (67.3)	17/52 (32.7)	26/52 (50)	8/52 (15.4)	18/52 (34.6)	20/52 (38.5)	32/52 (61.5)
	Cho et al. (11)	83	5.5 ± 2.6	1.5 ± 2.7	57.6 ± 10.0	33/83 (39.8)	50/83 (60.2)	25/83 (30.1)	58/83 (69.9)	NR	NR	NR
	Hwang et al. (12)	15	4.8 ± 2.7	3.2	67	NR	NR	NR	NR	NR	NR	NR
	Bhosale et al. (13)	11	8.4 ± 4.4	2.2 ± 1.7	71.2 ± 8.4	NR	NR	NR	NR	NR	NR	NR
	Seino et al. (14)	41	6 ± 1.8	NR	NR	24/41 (58.5)	22/41 (53.7)	18/41 (43.9)	23/41 (56.1)	NR	NR	NR
	Yang et al. (15)	80	4.3 ± 1.8	NR	54.7 ± 12.6	53/80 (66.3)	27/80 (33.8)	10/80 (12.5)	24/80 (30)	46/80 (57.5)	57/80 (71.3)	23/80 (28.8)
	Kang et al. (16)	4	4.3	4.8	72	3 (F)	1 (D)	4/4 (100)	NR	NR	2/4 (50)	2/4 (50)
	Kwon et al. (17)	52	5.5 ± 2.6	3.7 ± 3.6	62.8 ± 10.8	40 (F)	12 (D)	NR	NR	NR	NR	NR
	Bertagna et al. (18)	28	6.5 ± 4.6	3.7 ± 2.8	69.3 ± 10	15/28 (53.6)	13/28 (46.4)	9/28 (32.1)	19/28 (67.9)	–	20/28 (71.4)	8/28 (28.6)
	Brown et al. (19)	–	–	–	–	–	–	–	–	–	–	–
	Sahin et al. (20)	55	4.0 ± 1.0	2.3 ± 1.6	59.8 ± 8.4	12/55 (21.8)	28/55 (50.9)	NR	NR	NR	NR	NR
	Reesink et al. (21)	30	NR	NR	NR	NR	NR	NR	NR	NR	NR	NR
	Kim et al. (22)	3	NR	NR	NR	NR	NR	NR	NR	NR	NR	NR
	Conrad et al. (23)	–	–	–	–	–	–	–	–	–	–	–
	Lee et al. (24)	NR	NR	NR	NR	NR	NR	NR	NR	NR	NR	NR
	Makis et al. (25)	49	7.3 ± 5.3	2.7 ± 2.0	68.8 ± 9.9	33/49 (67.3)	7/49 (14.3)	NR	NR	NR	NR	NR
	Chetan et al. (26)	2	NR	NR	NR	NR	NR	NR	NR	NR	NR	NR
	Mannas et al. (27)	15	8.06	NR	69.9	NR	NR	NR	NR	NR	NR	NR
	Şahin et al. (28)	46	6.4 ± 1.9	1.08	62.3 ± 9.6	NR	NR	NR	NR	NR	NR	NR
	Lee et al. (29)	17	NR	NR	68.8 ± 6.1	NR	NR	NR	NR	NR	NR	NR
	Nieri et al. (30)	13	8.4 ± 4.9	8.8 ± 12.4	71.1 ± 7.6	NR	NR	NR	NR	NR	NR	NR
	Franklin et al. (31)	130	5.7	1.6	73	NR	57/130 (43.8)	NR	NR	NR	NR	NR
	Şam Özdemir et al. (32)	13	3.8	5.1	65	7 (F)	6 (D)	NR	NR	NR	NR	NR
Radiolabeled somatostatin analogues	Gossili et al. (33)	3	NR	NR	NR	NR	NR	NR	NR	NR	NR	NR

D, diffuse; F, focal; NR, not reported; NC, not calculable; SUVmax, maximal standardized uptake value.

#### 3.2.2 Somatostatin receptor PET/CT

Only one eligible study investigated incidental prostatic uptake on PET/CT performed with a radiolabeled somatostatin analogue (33). In that retrospective series, PIU was observed in 8 of 178 examinations (4.5%). Histology was available in 3 cases, none of which proved malignant, while the benign diagnoses included benign prostatic hyperplasia and other non-malignant conditions (33). Given the lack of additional homogeneous studies, this tracer was considered only in the qualitative synthesis.

#### 3.2.3 Overall study quality

Using the NIH Quality Assessment Tools, the overall methodological quality of the included literature was judged as moderate. The main sources of bias were the predominance of retrospective observational designs, the inconsistent use of histopathology as reference standard, incomplete reporting of some lesion-level variables, and the heterogeneous criteria used to select lesions for biopsy or follow-up. These limitations are typical of diagnostic evidence syntheses in this field and should be considered when interpreting pooled estimates and between-study variability.

### 3.3 Quantitative synthesis

#### 3.3.1 Pooled estimates of prevalence and malignancy

**Figure 2:**
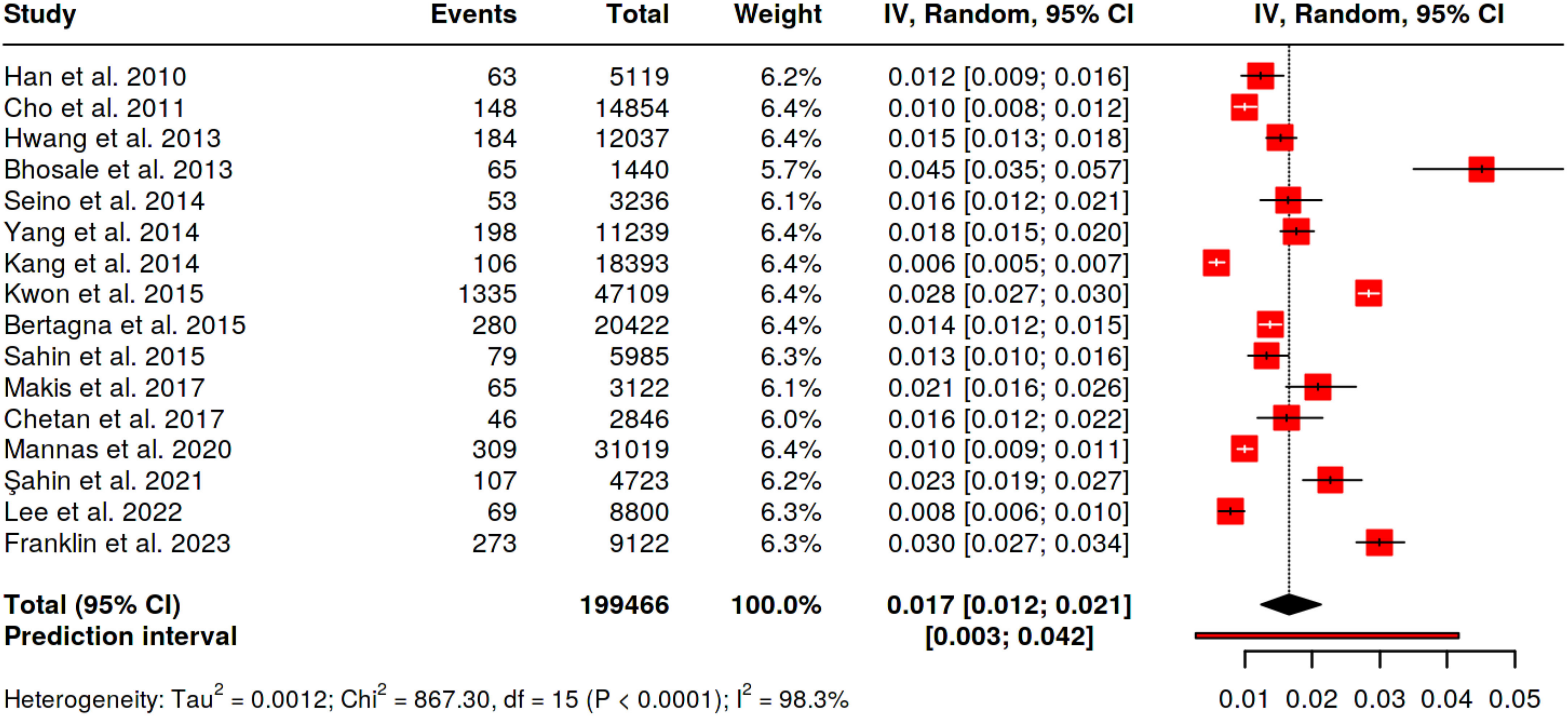
Pooled prevalence of PIU among [18F]FDG PET/CT.

In the analysable [18F]FDG series, the pooled prevalence of PIU among PET/CT examinations was 1.7% (95% CI, 1.2–2.1), as reported in **Figure 2**.

**Figure 3:**
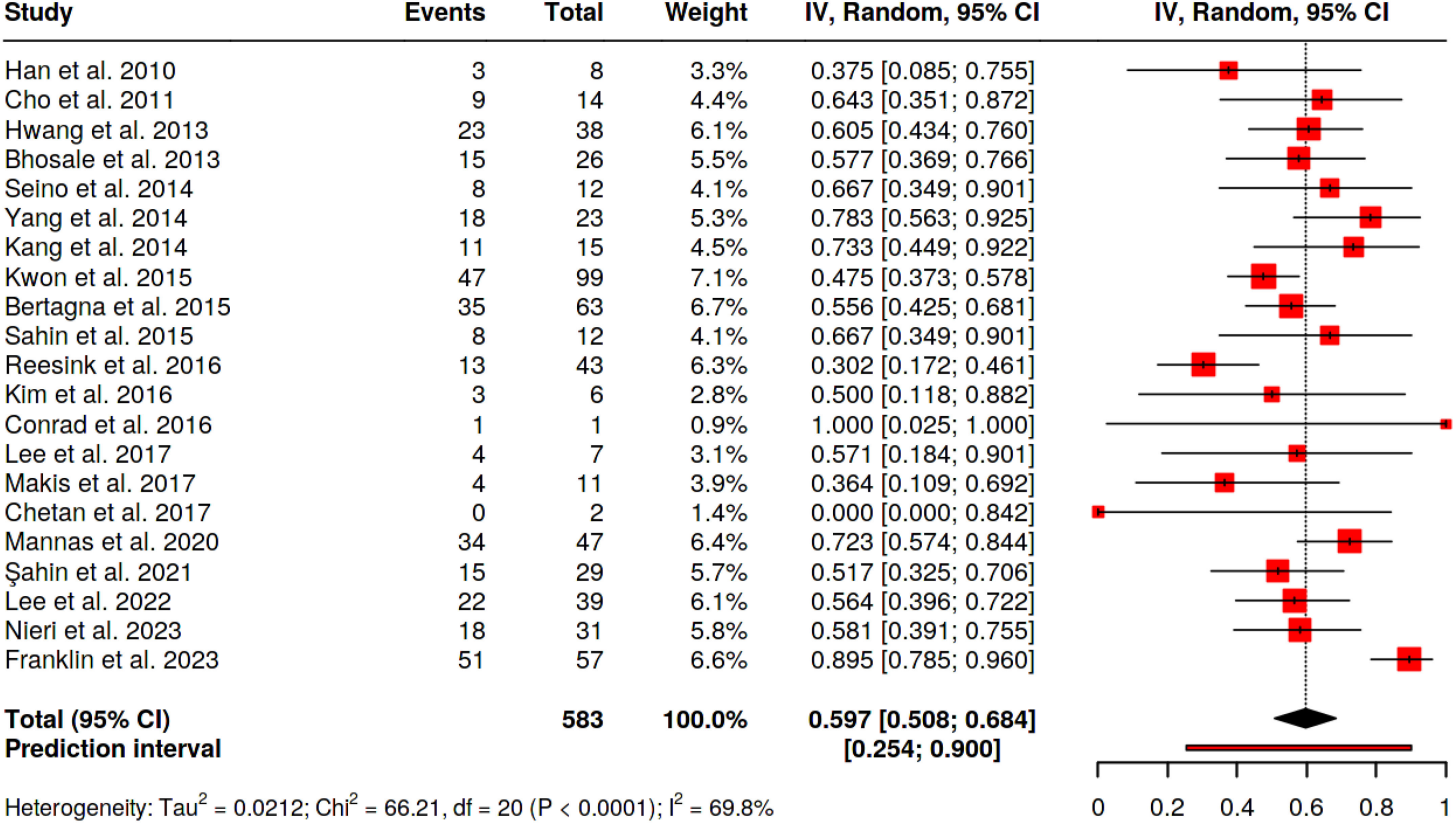
Pooled prevalence of malignant lesions among histologically verified PIUs at [18F]FDG PET/CT.

Among histologically verified PIUs, the pooled proportion of malignant lesions was 59.7% (95% CI, 50.8–68.4), as reported in **Figure 3**, indicating that more than half of the verified incidental lesions corresponded to malignancy. However, this estimate is subject to verification bias: biopsy was generally reserved for lesions considered clinically more suspicious, based on PSA level, uptake pattern, or physician judgment, and therefore the 59.7% figure should not be interpreted as the malignancy probability of all PIUs, but rather as the malignancy yield within a selected, suspicion-enriched subgroup.

**Figure 4:**
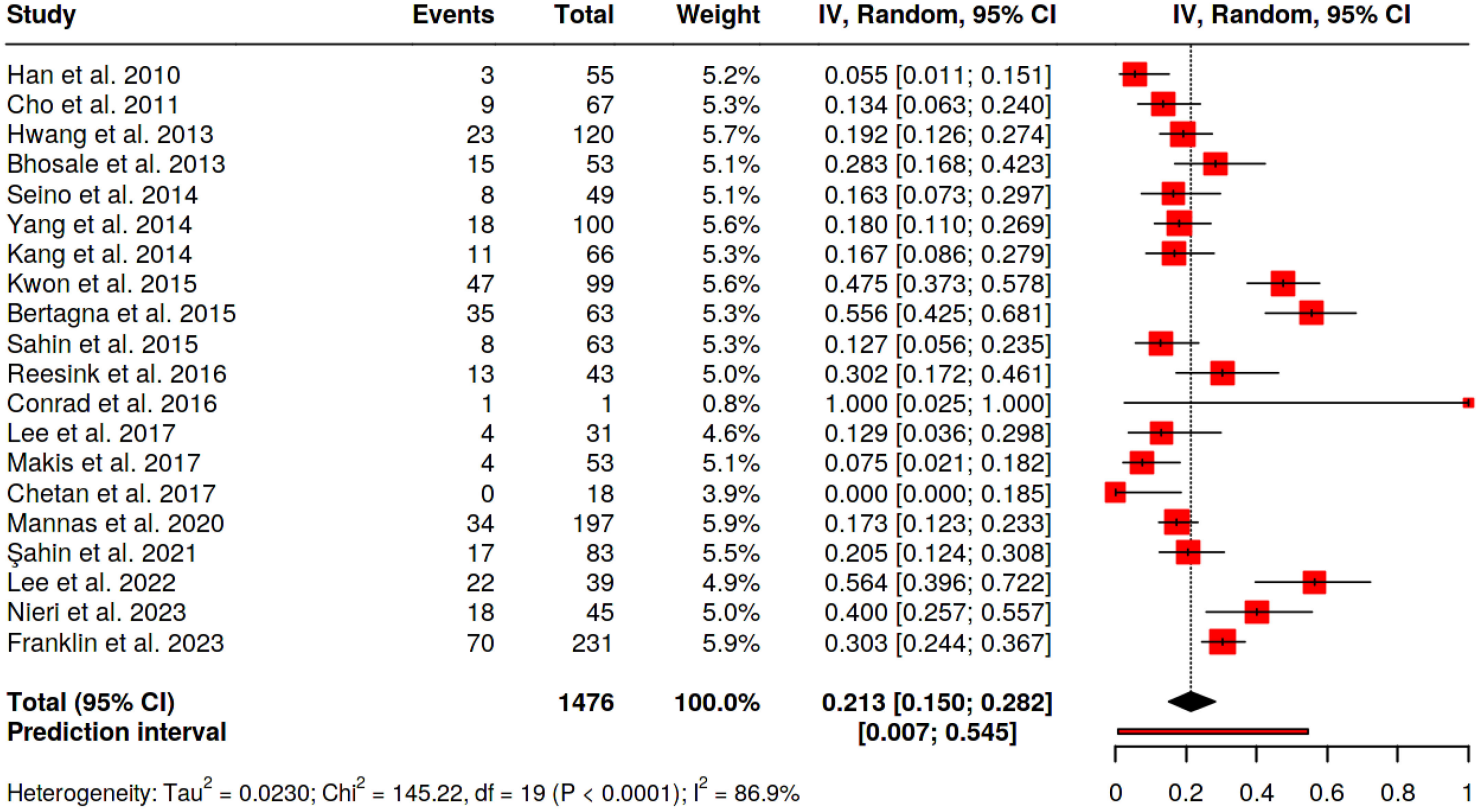
Pooled prevalence of malignant lesions among further evaluated PIUs at [18F]FDG PET/CT.

When the denominator was restricted to lesions undergoing further evaluation, the pooled prevalence of malignant PIU was 21.3% (95% CI, 15.0–28.2), as reported in **Figure 4**.

Altogether, these data indicate that incidental prostatic uptake is relatively infrequent on [18F]FDG PET/CT, but clinically relevant once identified and investigated (10–32).

#### 3.3.2 Quantitative correlates of malignancy

**Figure 5:**
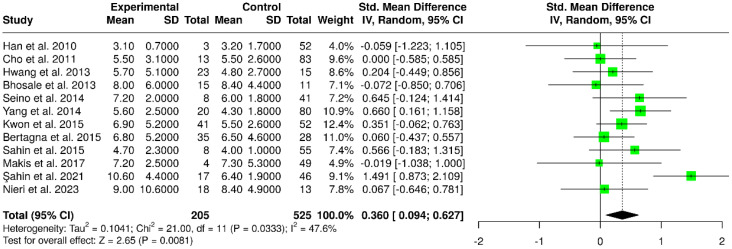
Forest plot about the SUVmax values.

Lesion-based meta-analysis in **Figure 5** showed that SUVmax was significantly higher in malignant than in benign PIUs, with a pooled standardized mean difference of 0.360 (95% CI, 0.094–0.627; I² = 47.6%), indicating a modest but statistically significant association between greater [18F]FDG avidity and malignancy. The heterogeneity was moderate, suggesting a reasonably consistent direction of effect across the contributing studies despite differences in cohort composition, lesion verification, and imaging setting. However, the overlap between benign and malignant lesions remained substantial, confirming that SUV-based metrics alone are insufficient for reliable lesion classification.

**Figure 6:**
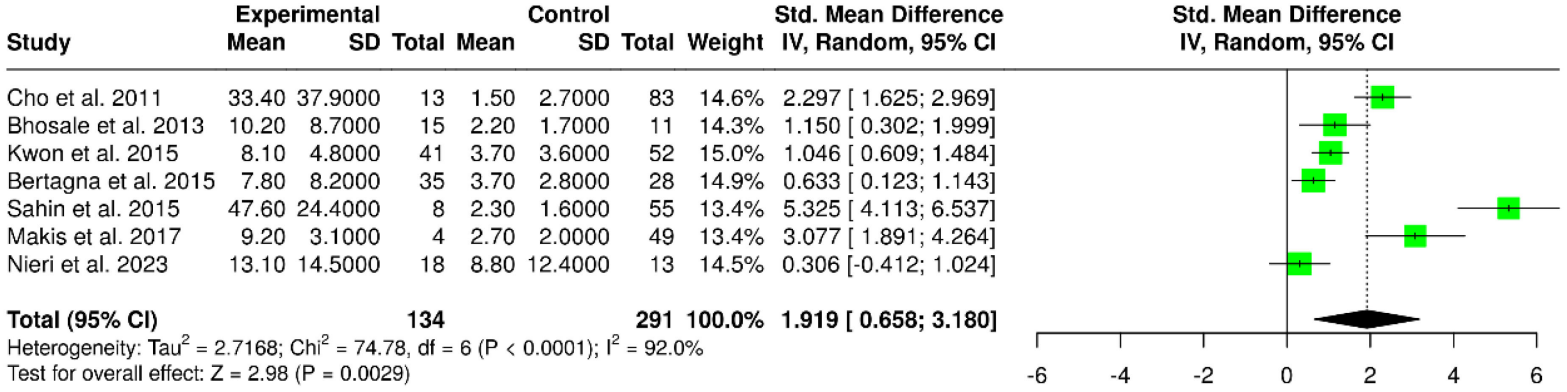
Forest plot of about the PSA values.

PSA values shown in **Figure 6** were also significantly higher in malignant lesions compared to benign findings, with a pooled standardized mean difference of 1.919 (95% CI, 0.658–3.180). However, this analysis showed very high heterogeneity (I² = 92.0%), implying marked between-study variability and suggesting that PSA, although clinically informative, is less reproducible across cohorts when pooled at the study level. This inconsistency likely reflects differences in biopsy indication, timing of PSA assessment, patient age distribution, and verification pathways rather than a lack of biological relevance.

**Figure 7:**
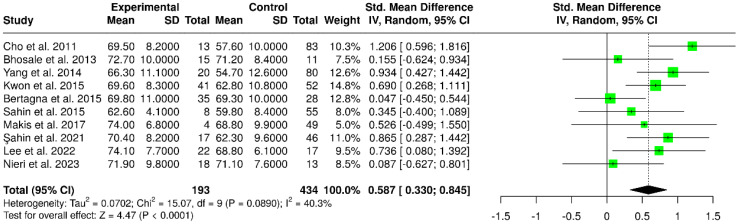
Forest plot of about age values.

Age was significantly higher in the malignant group than in the benign group (**Figure 7**), with a pooled standardized mean difference of 0.587 (95% CI, 0.330–0.845; I² = 40.3%), indicating a moderate association between older age and malignant PIU. The degree of heterogeneity was moderate, suggesting an overall concordant direction of effect across the included studies despite differences in cohort composition and diagnostic work-up. Although age may contribute to clinical risk stratification, its discriminatory role remains limited when considered in isolation and should therefore be interpreted together with imaging and biochemical findings.

#### 3.3.3 Uptake site and calcification pattern

**Figure 8:**
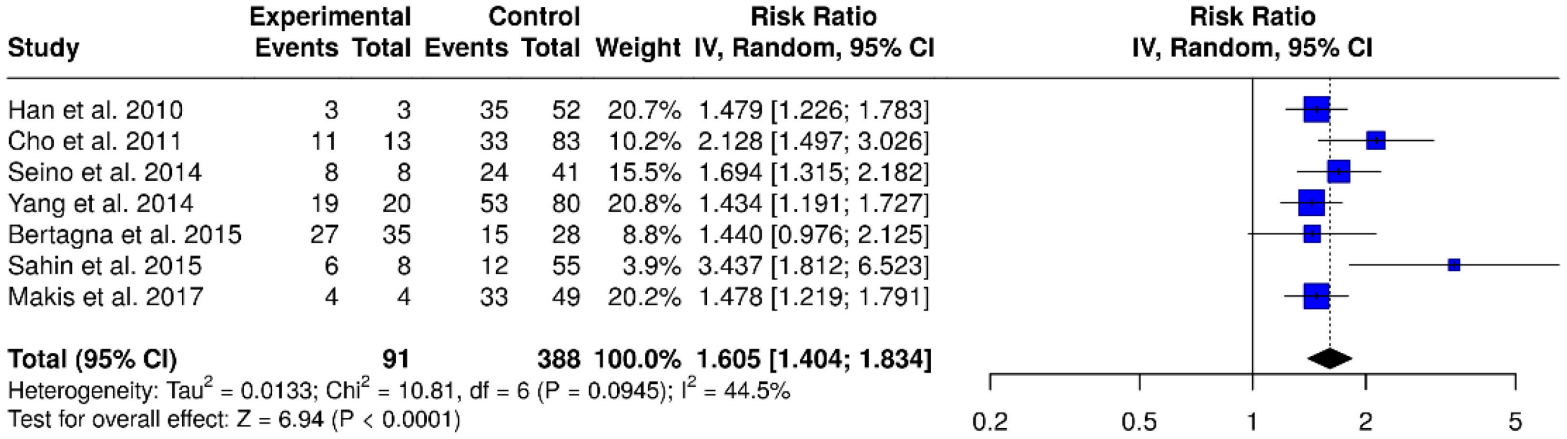
Forest plot on peripheral uptake.

Among topographic variables, peripheral uptake (**Figure 8**) was significantly associated with malignancy, with a pooled risk ratio of 1.605 (95% CI, 1.404–1.834; I² = 44.5%). This finding is clinically relevant because it supports the impression, already reported in several original series, that peripheral [18F]FDG uptake deserves greater diagnostic attention than central activity, which is more often related to benign prostatic conditions.

By contrast, central uptake was not significantly associated with malignancy, with a pooled risk ratio of 1.092 (95% CI, 0.691–1.725; I² = 63.5%). This analysis showed greater heterogeneity, reinforcing the limited specificity of central uptake as an isolated imaging marker.

Similarly, concordant calcification did not significantly differentiate malignant from benign lesions, with a pooled risk ratio of 0.769 (95% CI, 0.578–1.023; I² = 0%). Discordant calcification, on the other hand, was associated with a significantly lower pooled risk ratio of 0.353 (95% CI, 0.207–0.603; I² = 0%), suggesting a more frequent association with benign uptake patterns.

#### 3.3.4 Heterogeneity across analyses

The degree of heterogeneity varied substantially across the pooled analyses. Moderate heterogeneity was observed for SUVmax, age, and peripheral uptake, suggesting a reasonably consistent direction of effect across studies despite differences in patient selection, imaging protocols, and downstream verification pathways. In contrast, heterogeneity was high for PSA and for central uptake, indicating greater between-study variability and a lower degree of reproducibility for these parameters when considered at the study level. By comparison, the analyses based on calcification patterns showed no measurable heterogeneity, which suggests closer agreement among the contributing studies, albeit within a smaller evidence base.

Overall, these findings indicate that lesion location within the prostate and metabolic intensity are more stable correlates of malignancy than PSA alone, whereas PSA remains clinically informative but highly sensitive to differences in cohort composition and work-up strategy. This pattern is consistent with the retrospective and heterogeneous nature of the available literature and supports the interpretation of imaging, biochemical, and anatomical variables in combination rather than in isolation.

#### 3.3.5 Publication bias

**Figure 9:**
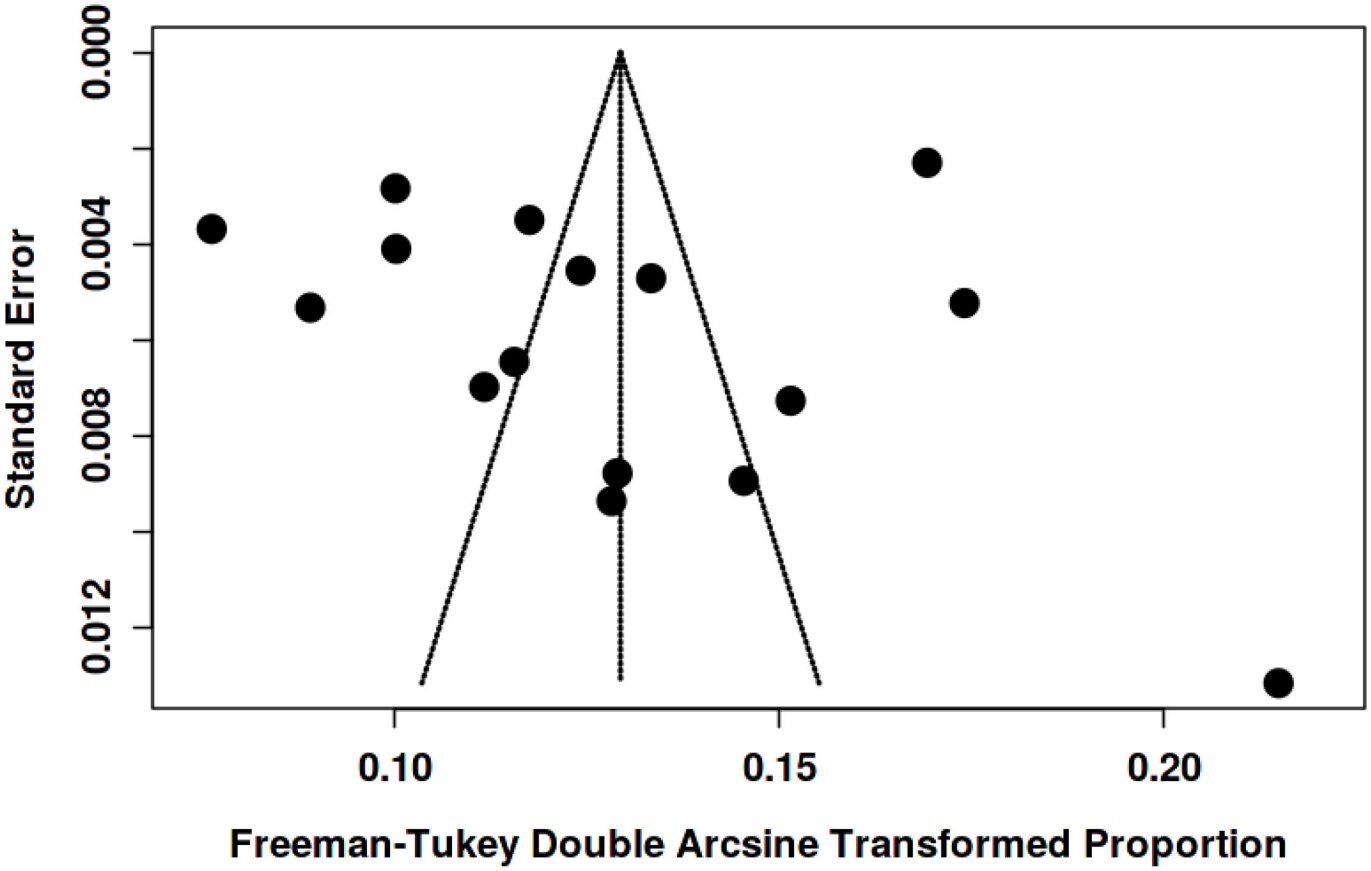
Funnel plot on PIU prevalence.

**Figure 10:**
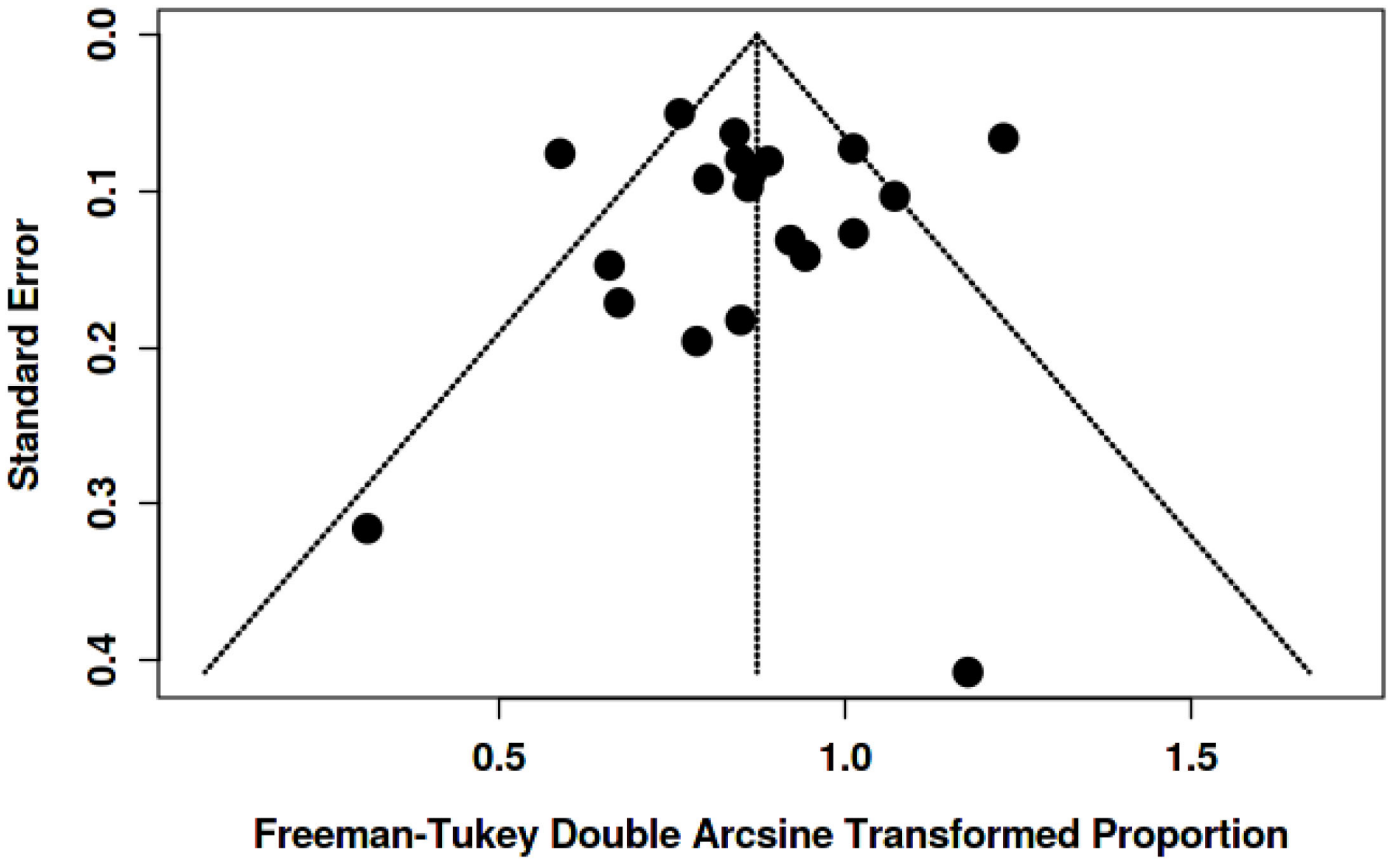
Funnel plot on malignancy among histologically verified PIUs.

**Figure 11:**
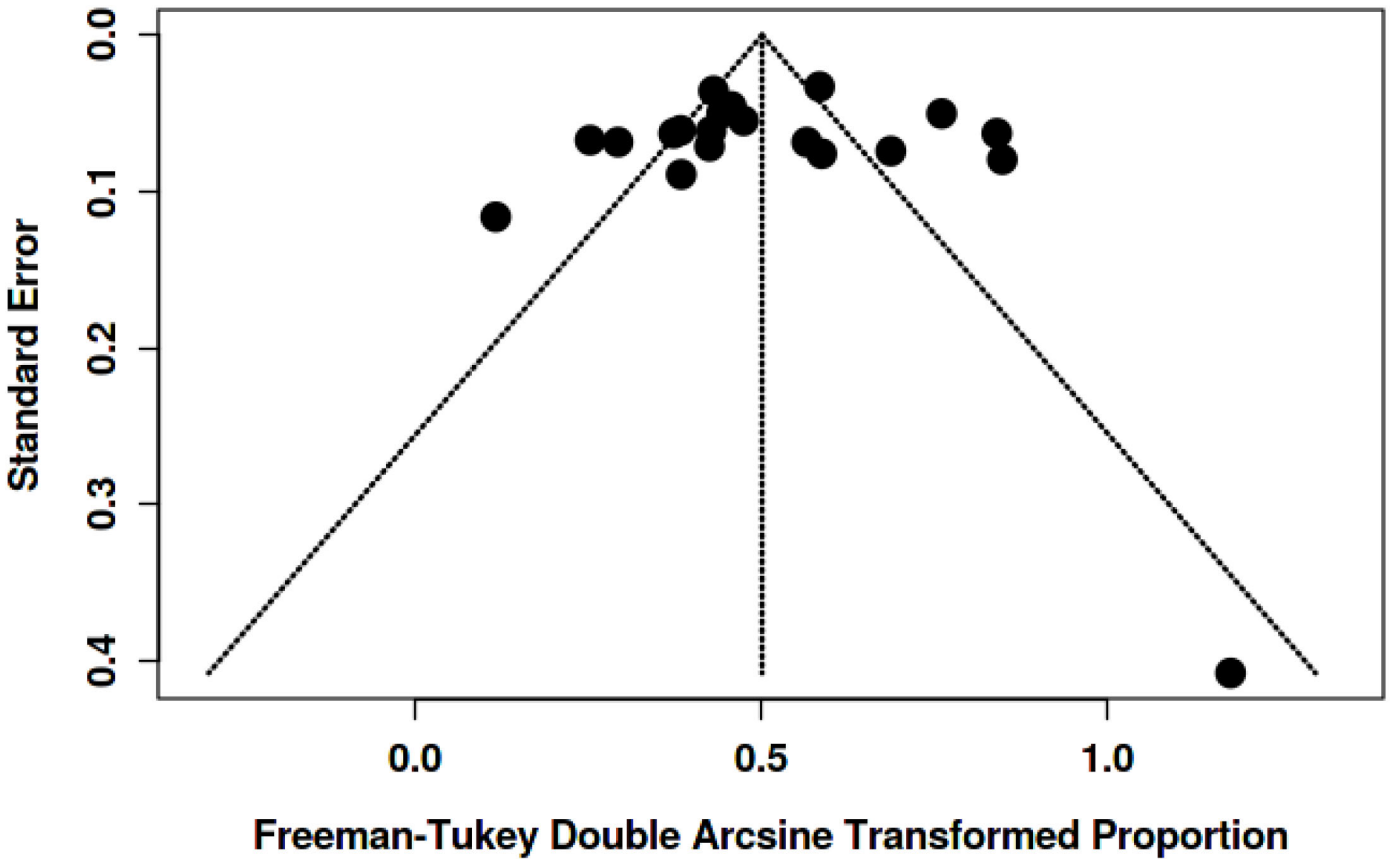
Funnel plot on malignancy among further evaluated PIUs.

Publication bias was evaluated visually using funnel plots and analytically using Egger’s regression test for the main proportion analyses. The funnel plots for PIU prevalence (**Figure 9**), histologically verified PIUs (**Figure 10**) and malignancy among further evaluated PIUs (**Figure 11**) were broadly symmetric, without a clear pattern of small-study effects or marked asymmetry. Consistently, Egger’s test was not statistically significant in any of the three analyses, with no evidence of funnel plot asymmetry for PIU prevalence (intercept: −0.21; 95% CI: −3.77 to 3.36; p = 0.911), malignancy among further evaluated PIUs (intercept: −0.22; 95% CI: −2.16 to 1.72; p = 0.826), or malignancy among histologically verified PIUs (intercept: −0.66; 95% CI: −9.81 to 8.5; p = 0.89).

Taken together, these results do not support the presence of a relevant publication bias in the principal pooled estimates. Nevertheless, this assessment should be interpreted cautiously, because funnel plot methods have limited power when the number of studies is modest and because retrospective diagnostic studies are inherently exposed to clinical and methodological heterogeneity, which can mimic or obscure asymmetry.

## 4 Discussion

### 4.1 Literature data

To our knowledge, the present study represents the most updated systematic review with meta-analysis specifically addressing incidental prostatic uptake (PIU) detected on PET/CT, with quantitative synthesis largely based on [18F]FDG studies and qualitative integration of the limited evidence available for other tracers. In order to reduce distortion of pooled estimates, case reports and non-original articles were excluded, and only studies providing extractable outcome data were considered for quantitative synthesis, in line with methodological recommendations for diagnostic evidence synthesis (16, 17). Our pooled results confirm that PIU on [18F]FDG PET/CT is relatively uncommon, with a prevalence of 1.7%, but clinically relevant once identified, since 59.7% of histologically verified lesions were malignant and 21.3% of further evaluated lesions ultimately corresponded to malignancy. It must be emphasised that these two estimates refer to clinically distinct denominators and carry different implications. The 59.7% malignancy rate applies exclusively to the subgroup undergoing histological verification, which was almost universally non-systematic and driven by clinical suspicion; this figure is therefore likely inflated by verification bias and should not be generalised to all PIUs. The 21.3% estimate, derived from the broader group undergoing any further clinical evaluation, represents a closer approximation of the malignancy yield among PIUs that prompted clinical action. Accordingly, the clinical response to PIU should be individualized rather than uniform, with greater attention to focal, peripherally located lesions and to cases associated with elevated PSA or other suspicious prostate-directed findings.

A major message emerging from this analysis is that the low frequency of PIU should not lead to underestimation of its clinical significance. The pooled prevalence observed in the present study is closely aligned with earlier meta-analytic estimates reporting values around 1.8%, supporting the stability of this finding across different study periods and patient populations (10–32). At the same time, the proportion of malignant lesions among verified incidental foci remains substantial, reinforcing the concept that unexpected prostatic uptake on [18F]FDG PET/CT should not be dismissed as a trivial or purely nonspecific finding (10–32). This aspect is particularly relevant in oncological patients, in whom PET/CT is usually performed for unrelated primary diseases and in whom clinically unsuspected second malignancies may still be encountered (1, 2).

From a biological and interpretative standpoint, the present results are consistent with the known limitations of [18F]FDG in prostate imaging. [18F]FDG uptake within the prostate is not specific for cancer and may be observed in adenocarcinoma, prostatitis, granulomatous inflammation, benign prostatic hyperplasia, and treatment-related changes, thereby explaining the overlap between benign and malignant incidental lesions reported across studies (10–32). This biological nonspecificity also helps explain why the pooled SUVmax difference, although statistically significant, remained moderate. In practical terms, SUV-based parameters may contribute to risk stratification but should not be regarded as sufficient in isolation for lesion classification, a conclusion that is consistent with both previous meta-analytic evidence and more recent management-oriented reviews (6).

The topographic distribution of uptake appears to be more informative. In our analysis, peripheral [18F]FDG uptake was significantly associated with malignancy, whereas central uptake was not. This observation is concordant with several original studies suggesting that focal peripheral activity is more suspicious for clinically significant prostate cancer than centrally located uptake, which more often reflects benign prostatic conditions (6, 10–32). By contrast, calcification-based analyses were less straightforward. Concordant calcification did not significantly separate benign from malignant lesions, whereas discordant calcification was more frequently associated with benign uptake patterns. Although these findings should be interpreted cautiously, they suggest that calcification pattern may be used as an ancillary feature rather than as a primary discriminator (6).

The PSA analysis warrants separate comment. Malignant lesions showed significantly higher PSA values, but the heterogeneity of this comparison was very high. This likely reflects differences across studies in biopsy thresholds, age distribution, timing of PSA measurement, referral setting, and diagnostic verification strategy, rather than a true absence of clinical relevance (10–32). Indeed, recent literature continues to support PSA as a useful complementary variable when interpreted alongside imaging findings and urologic evaluation rather than as an isolated marker. Similarly, age showed only a modest association with malignancy, reinforcing the concept that lesion-centered imaging and biochemical variables are more informative than demographic features alone (10–32).

Although the quantitative synthesis was largely restricted to [18F]FDG, the qualitative review also incorporated the small amount of non-FDG evidence available in the selected literature. The single eligible study based on radiolabeled somatostatin analogues did not identify malignant lesions among the histologically verified incidental prostatic foci, but the sample size was too limited to support tracer-specific conclusions (33).

Incidental prostatic tracer uptake may also be encountered using PET tracers beyond [18F]FDG, but these data remain too sparse and heterogeneous for formal pooling in the present framework. Therefore, the current conclusions should be interpreted primarily in relation to incidental PIU detected on [18F]FDG PET/CT.

#### 4.1.1 What to do with an incidental focus

The present findings support a structured and clinically pragmatic approach to incidental focal prostatic uptake on PET/CT. Because the pooled malignancy rate among verified lesions was substantial, incidental PIU should not be ignored, particularly when uptake is focal, peripherally located, or associated with elevated PSA or suspicious prostate-directed imaging findings (10–32). At the same time, the overlap in SUVmax values between benign and malignant lesions indicates that PET semiquantification alone should not guide management decisions.

In practical terms, additional evaluation should be individualized and may include serum PSA, digital rectal examination, urologic referral, mpMRI, and biopsy when clinically indicated. This strategy is consistent with the broader literature on incidental focal uptake at PET/CT, where the clinical challenge is to balance early detection of clinically significant malignancy against the risk of unnecessary invasive work-up (1, 2, 34). The present data suggest that integration of metabolic, topographic, and clinical information is preferable to reliance on any single PET-derived or laboratory parameter.

### 4.2 Limitations and suggestions for future research

This study has limitations. First, although the [18F]FDG evidence on PIU was broader than in earlier analyses (5), much of the literature remained retrospective, single-center, and methodologically heterogeneous, which inevitably affects the robustness of pooled estimates (10–32).

Second, the electronic literature search was restricted to PubMed/MEDLINE and the Cochrane Library, which together provide comprehensive coverage of the peer-reviewed nuclear medicine literature; manual reference checking of all included articles and relevant reviews was additionally performed to further strengthen retrieval completeness. The absence of a formal search in additional databases such as Embase or Scopus represents a limitation, although it is unlikely to have substantially affected retrieval given the highly specific nature of the topic.

Third, histologic verification was incomplete in many cohorts, introducing verification bias; this is not a peripheral concern but a central constraint on inference, since biopsy decisions were rarely systematic and were almost always driven by clinical suspicion. As a result, the 59.7% pooled malignancy rate among histologically verified PIUs is likely to overestimate the true malignancy risk of incidental prostatic uptake in unselected populations. A sensitivity analysis restricted to studies with predefined and systematic downstream work-up pathways was considered but could not be performed, as the majority of included studies did not report explicit biopsy criteria; this represents a fundamental limitation of the current evidence base and underscores the need for prospective studies with standardised work-up protocols.

Fourth, heterogeneity was substantial for several endpoints, especially PSA and some lesion-pattern analyses. Differences in study design, patient selection, prevalence denominator, biopsy policy, and reporting style likely contributed to the observed between-study inconsistency. In addition, some studies were informative for lesion characterization but not suitable for pooled prevalence analysis because they were based on selected cohorts rather than on unselected PET/CT populations. This issue was handled conservatively in the present review, but remains an intrinsic limitation of the currently available literature.

In closing, the evidence on PIU beyond [18F]FDG was very limited. It should be however acknowledged that some PET tracers are currently used for prostate cancer management in the clinical practice (e.g., prostate-specific membrane antigen ligands) and their uptake in the prostate gland cannot therefore be considered an incidental finding (35).

Future research should ideally rely on prospective, multicenter datasets with clearly reported denominators, standardized definitions of incidental uptake, and prespecified diagnostic pathways after lesion detection. Broader histopathologic verification, systematic reporting of PSA, mpMRI findings, lesion location, and calcification pattern would improve comparability across studies and refine risk stratification. Integration of radiomics, machine learning, and dedicated prostate imaging may also help identify which incidental lesions warrant biopsy and which may be safely observed, but these approaches still require robust external validation before routine clinical implementation.

## 5 Conclusions

In routine clinical practice, incidental prostatic uptake on [18F]FDG PET/CT is uncommon but clinically relevant, since a substantial proportion of verified lesions prove malignant. Among the variables explored, peripheral uptake, higher SUVmax, and higher PSA levels were more frequently associated with malignancy, whereas no single parameter was sufficiently accurate when considered in isolation. Accordingly, incidental prostatic uptake should prompt individualized correlation with clinical findings and prostate-directed assessment, including PSA testing, urologic evaluation, and mpMRI and/or biopsy when appropriate, particularly when uptake is focal or peripherally located.

Evidence beyond [18F]FDG remains limited. The few available non-FDG data, including somatostatin receptor-based imaging, are insufficient to support tracer-specific conclusions or management algorithms. Larger, methodologically standardized prospective studies are therefore needed to refine prevalence and malignancy-risk estimates, improve lesion-level risk stratification, and define more consistent diagnostic pathways for incidental prostatic uptake detected on [18F]FDG PET/CT.

## Data Availability

The original contributions presented in the study are included in the article/supplementary material. Further inquiries can be directed to the corresponding author.
